# Dual-wavelength UV-visible metalens for multispectral photoacoustic microscopy: A simulation study

**DOI:** 10.1016/j.pacs.2023.100545

**Published:** 2023-08-16

**Authors:** Aleksandr Barulin, Hyemi Park, Byullee Park, Inki Kim

**Affiliations:** a Department of Biophysics, Institute of Quantum Biophysics, Sungkyunkwan University, Suwon 16419, Republic of Korea; b Department of Intelligent Precision Healthcare Convergence, Sungkyunkwan University, Suwon 16419, Republic of Korea

**Keywords:** Multilayer metalens, Cascade metalens, Multispectral imaging, Ultraviolet, High NA, Photoacoustic microscopy

## Abstract

Photoacoustic microscopy is advancing with research on utilizing ultraviolet and visible light. Dual-wavelength approaches are sought for observing DNA/RNA- and vascular-related disorders. However, the availability of high numerical aperture lenses covering both ultraviolet and visible wavelengths is severely limited due to challenges such as chromatic aberration in the optics. Herein, we present a groundbreaking proposal as a pioneering simulation study for incorporating multilayer metalenses into ultraviolet-visible photoacoustic microscopy. The proposed metalens has a thickness of 1.4 µm and high numerical aperture of 0.8. By arranging cylindrical hafnium oxide nanopillars, we design an achromatic transmissive lens for 266 and 532 nm wavelengths. The metalens achieves a diffraction-limited focal spot, surpassing commercially available objective lenses. Through three-dimensional photoacoustic simulation, we demonstrate high-resolution imaging with superior endogenous contrast of targets with ultraviolet and visible optical absorption bands. This metalens will open new possibilities for downsized multispectral photoacoustic microscopy in clinical and preclinical applications.

## 1 Introduction

Photoacoustic microscopy (PAM) is a powerful imaging technique that addresses the long-standing challenge of high-resolution imaging of natural light absorption contrasts within living organisms [1], [2], [3], [4], [5]. This enables the visualization of various important intrinsic substances, including DNA/RNA and hemoglobin, that play crucial roles in several diseases and conditions. The cell nucleus, which houses the DNA genome, exhibits strong absorption of ultraviolet (UV) light. Morphological changes in cell nuclei, such as enlargement and folding of the nuclear envelope, are characteristic features of cancer cells. Therefore, PAM can aid in detecting and analyzing these changes, contributing to the understanding and diagnosis of cancer [6], [7], [8], [9], [10]. Hemoglobin, a key absorber in the visible spectral range, carries oxygen in the bloodstream. Hypoxia is a fundamental characteristic of the cancer cells. PAM can provide valuable information on cancer-related processes by mapping the distribution and oxygen saturation of hemoglobin [11], [12], [13], [14], [15]. Given that these two areas represent the main research streams in PAM, combining both capabilities within a single system would offer considerable advantages in terms of space and cost efficiency. Cao and Hu et al.'s work, which integrates these two system schemes, is a noteworthy example. They developed a novel optical-acoustic objective, allowing the acquisition of UV–visible-near-infrared PA images using a single system, and emphasized its potential for future advancements [16]. Additionally, simultaneous imaging of multiple endogenous optical absorbers using PAM holds great promise for both basic research and clinical applications in various diseases. For example, the UV–visible PAM can simultaneously serve as virtual histopathology and accurate observation of surrounding blood vessels in Mohs surgical procedures [17]. However, the development of high-resolution dual-wavelength PAM that covers from UV to visible wavelengths is challenging owing to issues such as chromatic aberrations in optical systems [16].

Metasurface devices are ubiquitous because they enable the creation of lenses [18], [19], [20], [21], dispersive elements [22], [23], or holograms [24], [25], [26], [27], [28] comprising two-dimensional arrangements of subwavelength structures. In particular, metalenses have become increasingly popular miniaturized imaging systems that exhibit focusing performance competitive with bulky and expensive high numerical aperture (NA) objective lenses [29], [30]. Among the known designs, multilayer metalenses enable an additional vertical degree of freedom to tailor incident electromagnetic fields in a two-dimensional space for achromatic focusing at several wavelengths [31], [32], [33] or diffraction-limited focusing at oblique incident angles [34]. Multiwavelength transmissive metalenses have multiple prospective applications in fluorescence microscopy, virtual reality, and digital projections [32]. Multilayer metalenses have been shown to correct two and three wavelengths in the visible [33] and infrared (IR) [31], [32] spectral ranges; however, designing UV–visible and high numerical aperture metalenses could pave the way for PAM applications with endogenous optical absorption contrast of DNA/RNA (266 nm) and hemoglobin (532 nm) [16], [35], [36]. Diffractive lenses recently demonstrated promising performance in PAM with elongated depth of focus [37], [38]. However, the phase modulation by those diffractive elements was typically binarized which would make the design of high NA and achromatic lenses problematic [39]. Several high refractive index dielectric materials have been utilized for UV transmissive metasurfaces, *for example,* niobium oxide, zinc oxide, and aluminum nitride down to near UV (355 nm) [40], [41], and silicon nitride, zirconium oxide, and hafnium oxide down to deep UV (266 nm) [40], [42], [43]. Recently, the first demonstration of extreme UV light focusing has been reported, in which a transmissive metalens operates via vacuum guiding in cylindrical hole arrangements milled in crystalline silicon films [44]. Hafnium oxide metalenses exhibit remarkable focusing performance at single wavelengths in deep UV [45] owing to their negligible absorption coefficient above 250 nm. Theoretical UV transmissive metalens designs proposed earlier provide achromatic focusing at several wavelengths [46], [47]; however, the operating wavelength range lies within the UV band and does not extend to the visible range. Reflective achromatic metalenses have been theoretically proposed to operate in the UV–visible range [48], although they are barely applicable to conventional PAM.

Here, we propose a numerical design of a transmissive achromatic metalens operating at deep-UV (266 nm) and visible (532 nm) wavelengths for PAM applications. We employed a two-layer metalens with HfO_2_ cylindrical nanopillars embedded in a silica-on-glass film and HfO_2_ cylindrical nanopillars of different heights and independent diameters stacked on top. The proposed design and nanostructure geometries are feasible for current electron-beam lithography solutions [32], [49], [50], [51]. The designed metalens exhibited a nearly perfect focus match (<20 nm focal length difference) for 266 and 532 nm wavelengths and diffraction-limited performance at a high NA = 0.8. The metalens NA appeared to be substantially higher than that of commercially available UV air-immersion objective lenses [52]. Extending multilayer lens operation to the UV range opens unique opportunities for multispectral PAM applications. Whereas sophisticated catadioptric objective lenses may enable high NA and deep-UV operation, yet, they fail to provide perfect chromatic aberration correction in a broad wavelength range [53]. We evaluate the lateral resolution of metalens-based UV–visible PAM using a three-dimensional (3D) photoacoustic (PA) simulation. Furthermore, we numerically confirmed that metalens-based UV–visible PAM incorporating a spectral unmixing technique was capable of distinguishing targets with distinct absorption coefficients $\mu_{a}$ for UV and visible light [54], [55], [56]. We note that the ultra-compact two-layer metalens structure has a 30 µm diameter and only 1.4 µm thickness that could perfectly fit into handheld [57], [58], pen-type [59], [60], and intravascular PAMs [61]. We envision that the designed metalens will pave the way for the development of portable multispectral PA devices for biological applications.

## 2 Methods

### 2.1 Overall simulation strategies

**Fig. 1:**
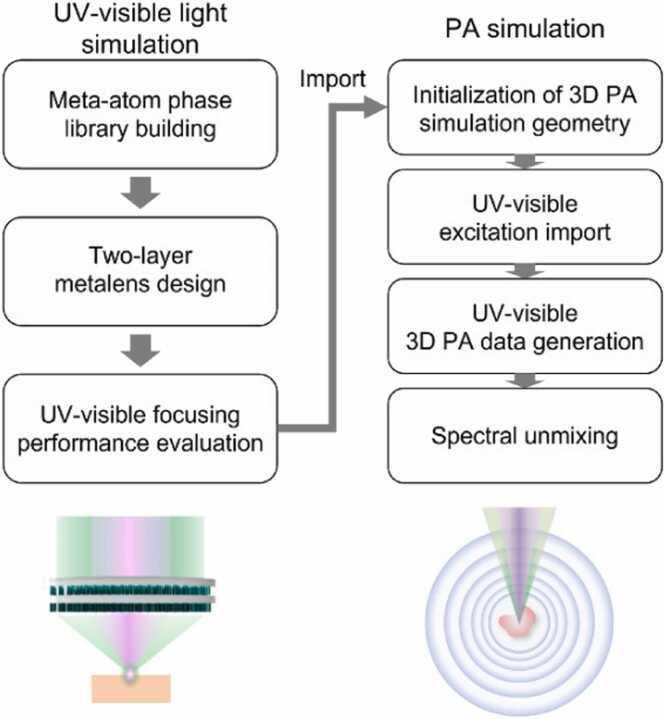
Proposed strategy to numerically assess two-wavelength multilayer metalens applicability to PAM. The arrows follow the simulation strategy workflow.

Fig. 1 shows the scheme of our simulation strategy to design a metalens and import the field intensity data in the focal volume for the numerical analysis of the PA response. The designed multilayer metalens operated as a dual-wavelength achromatic lens at 266 and 532 nm. The micrometer-thick nanostructure arrangement of the metalens made it an ultracompact and portable device to excite a PA response from targets that exhibit spectrally different absorption bands, such as cell nuclei (DNA/RNA at 266 nm) and blood vessels (hemoglobin at 532 nm). In the first step, a meta-atom library was built to attain phase control over transmitted light at both wavelengths, and then a two-dimensional circular arrangement of meta-atoms is assembled for diffraction-limited and high-NA focusing, simultaneously. The achromatic metalens focusing was determined based on FDTD numerical simulations. The resulting intensity field profiles were imported into the k-Wave toolbox in MATLAB for 3D PA simulations. Based on the PA response, we numerically quantified the lateral resolution and imaging of the targets with two DNA/RNA- and hemoglobin-mimicking phantoms using the same metalens platform. In addition, because both phantoms exhibited high UV light absorption, only the components of each phantom were extracted by applying the spectral unmixing technique [55], [62].

### 2.2 Metalens building blocks

**Fig. 2:**
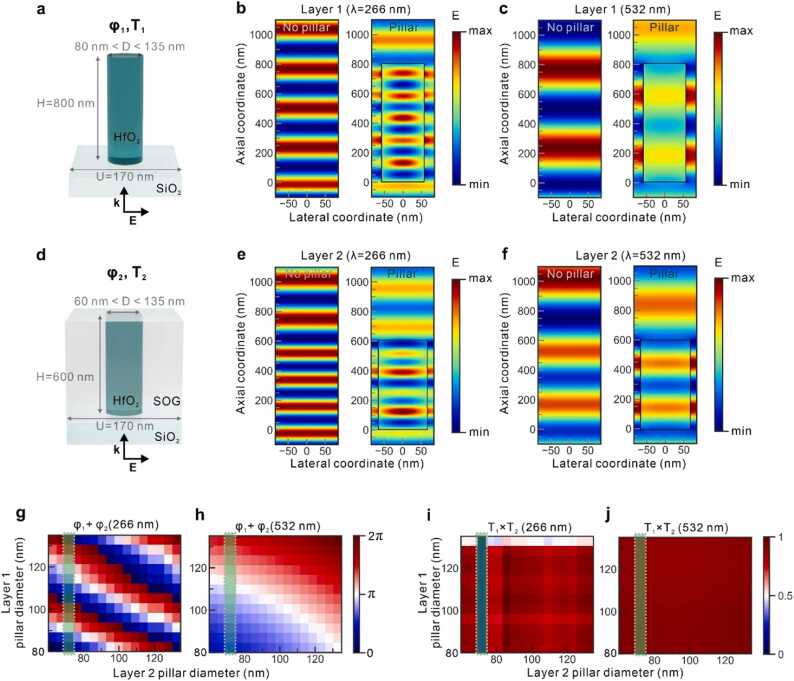
Two-layer metalens design optimization. (a, d) Top (Layer 1) and bottom (Layer 2) layers of cylindrical nanopillar geometries employed as unit cells for the metalens design, respectively. (b, c) Electric field showing the phase shift upon the propagation through a pillar of Layer 1 in the air (right) as compared with the reference in the absence of the pillar (left) at 266 nm and 532 nm, respectively. The diameter of the pillar in the air is D = 115 nm. (e, f) Electric field showing the phase shift upon the propagation through a pillar of Layer 2 embedded in SOG (right) as compared with the reference SOG layer in the absence of the pillar (left) at 266 nm and 532 nm, respectively. The diameter of the pillar in SOG is D = 135 nm. (g, h) Calculated overall wrapped phase maps as a function of Layer 1 and Layer 2 pillar diameters for 266 nm and 532 nm wavelengths, respectively. Data retrieved from the unit cell simulations for both layers are phase and transmission variations (φ, T). (i, j) Calculated overall transmission maps as a function of Layer 1 and Layer 2 pillar diameters for 266 nm and 532 nm wavelengths, respectively. The overall phase and transmission data of each meta-atom are computed as a sum of phases and a product of transmission values of the top and bottom pillars. The Layer 2 pillar diameter of 70 nm (marked by green dashed framed shading) that sits in a transmission dip at 266 nm wavelength is removed from the metalens building library.

FDTD simulations of the meta-atom phase shifts and metalens focusing were performed using Ansys Lumerical FDTD R1.4 software. The complex refractive indices of HfO_2_ nanostructures were adopted from [45]. Fig. 2 shows the meta-atom library building to enable point-by-point control over the phase of the transmitted light. HfO_2_ was selected as a meta-atom material because it exhibited a modest refractive index and low losses in deep UV light, enabling highly efficient UV light focusing by a single-wavelength metalens [45]. The HfO_2_ thin film bandgap is reported to be 5.7 eV (λ = 218 nm) [45], which is sufficiently higher than the absorption energy of DNA/RNA constructs or proteins in the UV. We investigated meta-atom combinations (Fig. 2a, d) with a top layer (Layer 1) of the HfO_2_ cylindrical nanopillar in air and a bottom layer (Layer 2) of the HfO_2_ cylindrical nanopillar embedded in silica-on-glass (SOG). The SOG and substrate refractive indices were set to SiO_2_-Palik. In addition to the horizontal degrees of freedom utilized via the pillar diameter selection, the two-layer meta-atom structure enabled an extra vertical degree of freedom to set the required phase profiles for achromatic lensing at wavelengths of 266 and 532 nm, simultaneously. The cylindrical geometry of meta-atoms supported polarization-independent focusing. The unit cell size (*U*) was maintained at 170 nm, which was approximately equal to the Nyquist sampling criterion threshold (U<λ/2NA) for the metalens design parameters of NA = 0.8 and *λ* = 266 nm [18]. The chosen nanopillar geometries complied with the electron beam lithography capabilities that typically required a (i) nanostructure height below 1 µm, (ii) minimal pillar diameter of 50 nm, (iii) pillar aspect ratio below 10, (iv) pillar diameter accuracy of 5 nm, and (v) large interpillar distance. To generate a fabricable metalens structure, the heights of the Layer 1 and Layer 2 nanopillars were fixed at 800 and 600 nm, respectively. These height values were chosen to cover the 2π phase modulation in each cascade meta-atom. The propagation phase modulation depends linearly on the height as follows [63]: $\varphi =k_{0}n_{\mathrm{eff}}h$, where *k*_*0*_ is the wavenumber in vacuum, *n*_*eff*_ denotes the effective refractive index, *h* is the meta-atom height. Therefore, any height combinations are expected to yield correct metalens performance, as long as the height values do not sit in transmission dips (Fig. S1). The potential fabrication process can include the following steps. The HfO2 etching process is implemented with the use of Damascene lithography [45]. A SOG layer can be spin-coated after the lithography etching process [64], [65], [66]. The second metasurface layer may be deposited via a subsequent e-beam lithography and HfO_2_ etching process. A similar strategy has been used for UV–visible hologram generators [43].

A meta-atom library comprising the phase and transmission information was built by simulation sweeps of pillar diameters from 80 to 135 nm for Layer 1 and from 60 to 135 nm for Layer 2 with 5 nm steps. The HfO_2_ meta-atoms were shown to induce a phase shift of the transmitted light through both Layer 1 (Fig. 2b, c) and Layer 2 (Fig. 2e, f) at wavelengths of 266 and 532 nm. Electromagnetic waves passing through the nanopillars clearly underwent a shift in wave oscillations, illustrating phase tunability at both UV and visible wavelengths. The phase of each possible vertical two-pillar cascade represented the superposition of the phase shifts of Layer 1 (φ_1_) and Layer 2 pillars (φ_2_) [32], [33]. Single-pillar layers strive to provide a 2π phase span at a visible wavelength of 532 nm owing to the low refractive index and limited diameter range. Nevertheless, the extra vertical degree of freedom empowered by two-pillar stacking unlocks the full 2π phase control at 532 nm via the sum of phase shifts of each layer. Thus, the proposed pillar cascade of HfO_2_ covers nearly three 2π spans at a wavelength of 266 nm and one 2π span at 532 nm (Fig. 2g, h). In principle, adding an extra metasurface layer could unlock a third working wavelength operation, for instance, in the infrared spectral range, where deep tissue PA imaging is more favorable. We show that a three-layer cascade can enable full 2π phase modulation at wavelength of 744 nm (Fig. S2), where breast cancer treatment can be visualized in vivo [67]. The refractive index of HfO_2_ appears to be too low to gain complete phase control of wavelengths beyond 1 µm. Choosing other materials with a higher refractive index may circumvent the obstacle, however, utilization of UV non-transparent material will lead to further reduction of the focusing efficiency in the deep UV range.

We verified that high near-field transmission values were maintained for each pillar structure and retrieved combinations of transmission value products for all two-pillar cascade combinations. The simulated near-field transmission maps (Fig. 2i, j) were free of broad transmission dips and yield values above 0.8 nearly for all employed meta-atoms at both wavelengths. Solely 70 nm pillars in SOG yield low transmission at 266 nm. Therefore, cascade options involving those pillars were excluded from the design of the metalens arrangement.

### 2.3 Simulation of 3D UV–visible PA data generation

**Fig. 3:**
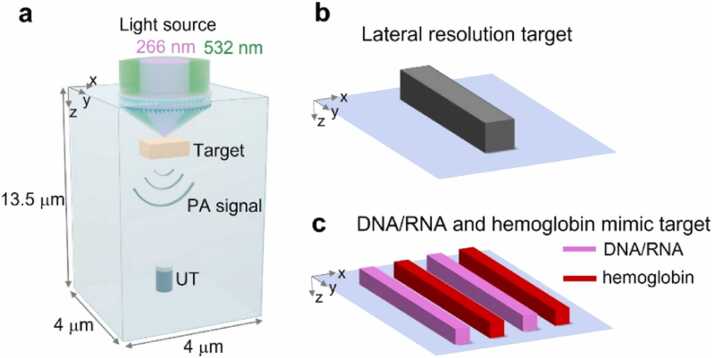
Three dimensional PA simulation initialization. (a) 3D PA simulation region. (b) Single cuboid target for the lateral resolution measurement and (c) four cuboid targets for the 3D PA imaging of DNA/RNA- and hemoglobin-mimicking phantoms. PA, photoacoustic; UT, ultrasound transducer.

We employed the k-Wave toolbox in the MATLAB environment to conduct 3D simulations of the UV–visible PA images. UV–visible excitation via the metalens was used as the light source in the simulations. The k-Wave toolbox was exclusively used to generate the PA data, whereas general MATLAB functions were employed for subsequent image processing and reconstruction tasks. To expedite the computation, the PA simulations were performed on a high-performance NVIDIA RTX 3060 graphics processing unit (GPU) that boasts 3584 CUDA cores and 12 GB RAM. The simulation was initialized by generating 3D geometry and importing metalens-based UV and visible light sources into a MATLAB workspace. The simulation space was set to 4 × 4 × 13.5 µm with 357 × 357 × 568 voxels. A perfectly matched layer was used to absorb the outgoing waves from the target, whereas the internal echoes from the surface were blocked. A single-element ultrasound transducer was placed at 0.97 µm away from the center of a target in the Z-direction. The center frequency of the transducer was set to 40 MHz with a 70% bandwidth. The medium was water and was assumed to be acoustically homogenous and lossless (Fig. 3a). To measure the lateral resolution, a single cuboid target was set to dimensions of 45 × 500 × 45 nm^3^ along the X-Y-Z direction (Fig. 3b). We set the initial pressure distribution in binary form in the phantom. To validate the UV–visible PA image generation using the metalens, we mimicked DNA/RNA and hemoglobin phantoms (Fig. 3c). The phantom consisted of four elongated cuboids, with pink cuboids mimicking optical absorption coefficient $\mu_{a}$ of DNA/RNA and red cuboids mimicking that of hemoglobin. We used normalized values by referring to DNA/RNA and hemoglobin $\mu_{a}$ in previous studies [68], [69], [70].

Each cuboid was configured with dimensions of 45 × 4000 × 45 nm^3^ along the X-Y-Z direction. Furthermore, a separation distance of 404 nm was maintained between the adjacent cuboids.

## 3 Results

### 3.1 Simulation results of the UV–visible metalens

**Fig. 4:**
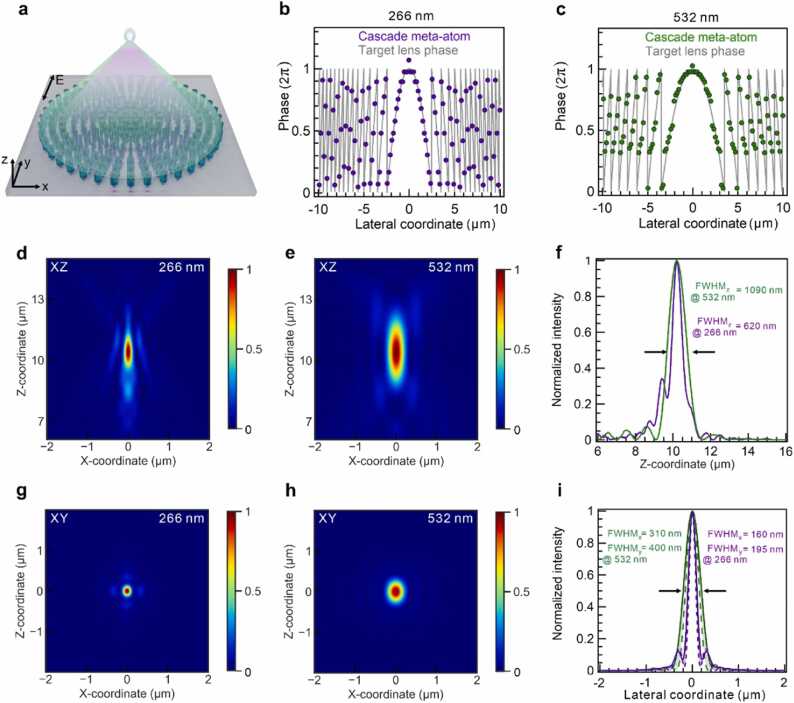
Simulated UV–visible metalens focusing performance. (a) Schematic two-layer metalens focusing of 266 and 532 nm light at the same focal length. The diameter of the metalens is 30 µm. (b, c) Target and design phase profiles of cascade meta-atoms in the metalens at 266 and 532 nm wavelengths, respectively. (d, e) Axial field intensity profiles for both wavelengths. Z-coordinate origin is set at the top surface of the metalens. (f) Line profiles along the z-axis for both wavelengths. The intensity peaks exist at nearly identical focal distances with the focal mismatch below 20 nm. (g, h) Lateral field intensity profiles for both wavelengths taken in the focal plane and (i) the corresponding line profiles along the x- and y-axes for both wavelengths.

Fig. 4a depicts the achromatic UV–visible two-layer metalens structure and light focusing at wavelengths of 266 and 532 nm. The target wavelength-dependent phase profile exhibits a hyperbolic behavior, as follows:

(1) $$\varphi_{\mathrm{target}}\left(\lambda ,x,y\right)=-\frac{2\pi }{\lambda }\left(\sqrt{x^{2}+y^{2}+f^{2}}-f\right)$$

Here, *x* and *y* denote the lateral Cartesian coordinates of the meta-atoms, and *f* denotes the desired focal length. The design NA is encoded in the focal length parameter as: $f=r_{0}/\tan(\arcsin\left(\mathrm{NA}\right))$ with $r_{0}$ being the radius of the metalens.

The achromatic focusing of a plane wave was achieved by adjusting the phase shift induced by the cascade units to the wrapped target phase at both wavelengths. The two-pillar cascade combination was selected via a brute force search to minimize the phase error function determined as follows [32]:

(2) $$\Delta^{\mathrm{ij}}\left(x,y\right)=\left|\varphi_{1}^{i}\left(266\mathrm{nm}\right)+\varphi_{2}^{j}\left(266\mathrm{nm}\right)-\varphi_{\mathrm{target}}\left(266\mathrm{nm},x,y\right)+2\pi n\right|+\left|\varphi_{1}^{i}\left(532\mathrm{nm}\right)+\varphi_{2}^{j}\left(532\mathrm{nm}\right)-\varphi_{\mathrm{target}}\left(532\mathrm{nm},x,y\right)+2\pi m\right|$$

Here, $\varphi_{1}^{i}$ and $\varphi_{2}^{j}$ represent the phase shifts of *i*-th and *j-*th pillars in meta-atom libraries for Layer 1 and Layer 2, respectively, and *n* and *m* represent integers that wrap the target phase to a 2π span. As the brute force search scans through all possible combinations, it finds the minimal error function value that can be considered as a global minimum. The metalens with an orthogonal array arrangement was built point-by-point by adding an optimal two-pillar combination in each unit cell of 170 nm. The diameter of the designed metalens was set to 30 µm such that the simulation requirements remained within the computational power of our workstation. To highlight the advantages of the metasurface as a flexible platform to tailor the EM field, we designed a metalens with a numerical aperture of 0.8, which was higher than that of commercially available UV air-immersion objective lenses [52]. The phase shifts of the designed cascade meta-atoms well matched the wrapped target phase maps of a lens with an NA = 0.8 (Eq. 1), at both wavelengths, as shown in Fig. 4b, c. Next, we simulated the focusing of a linearly polarized plane wave at the two designed wavelengths (Fig. 4d-i). The apparent working distance of the metalens was around 10.4 µm, appropriately corresponding to the design NA of 0.8, and the focal spot mismatch between two wavelengths appeared to be within 20 nm (Fig. 4f). The axial beam FWHMs were 620 and 1090 nm for wavelengths of 266 and 532 nm, respectively. The lateral beam FWHM existed in the range of 160–195 nm at λ = 266 nm and 310–400 nm at λ = 532 nm, depending on the linear polarization orientation. Based on the retrieved intensity distribution, we computed metalens Strehl ratios by normalizing lateral intensity cuts to the Airy patterns of an ideal lens ($I(x)=4∙{\left[J_{1}\left(2\pi ∙\mathrm{NA}∙x/\lambda \right)/(2\pi ∙\mathrm{NA}∙x/\lambda )\right]}^{2}$, where *J*_*1*_ represents a Bessel function of the first kind of order one) with the same area under the curve. The Strehl ratios determined from *x*-axis horizontal cuts were 0.83 and 0.98 at λ = 266 nm and λ = 532 nm, respectively. These values exceeded 0.8, the industry-based Strehl ratio threshold validating the diffraction-limited performance [71]. The focusing efficiency of the metalens was determined by dividing the source power passing through a narrow square monitor of 4 × 4 µm^2^ placed at the focal spot by the total incident power. The focusing efficiencies were 16.3% at λ = 266 nm and 26.1% at λ = 532 nm. The relatively low focusing efficiency could be related to the losses of two-pillar stacking in the cascade as well as the high NA of the metalens made of a material with a relatively low refractive index [32], [40], [72]. Nevertheless, the typical independently reported focusing efficiency of an RGB two-layer metalens with NA= 0.8 can be as low as 13% in the green and blue spectral ranges [33]. The determined near-field transmission through our metalens was 62.7% at λ = 266 nm and 91.7% at λ = 532 nm. We estimate the laser-induced damage threshold of the HfO_2_ metalens following a recently reported quantitative method [73]. The resulting energy damage threshold amounts to 49 J/cm^2^ at 266 nm and 460 J/cm^2^ at 532 nm. Assuming a 10 ns laser pulse width for PA imaging, the peak power density threshold would appear to be 4.9 GW/cm^2^ and 46 GW/cm^2^ for the respective wavelengths (see details in Supplementary information section S3). These values are comparable to a typical optical fiber damage threshold [74].

### 3.2 3D PA simulations

**Fig. 5:**
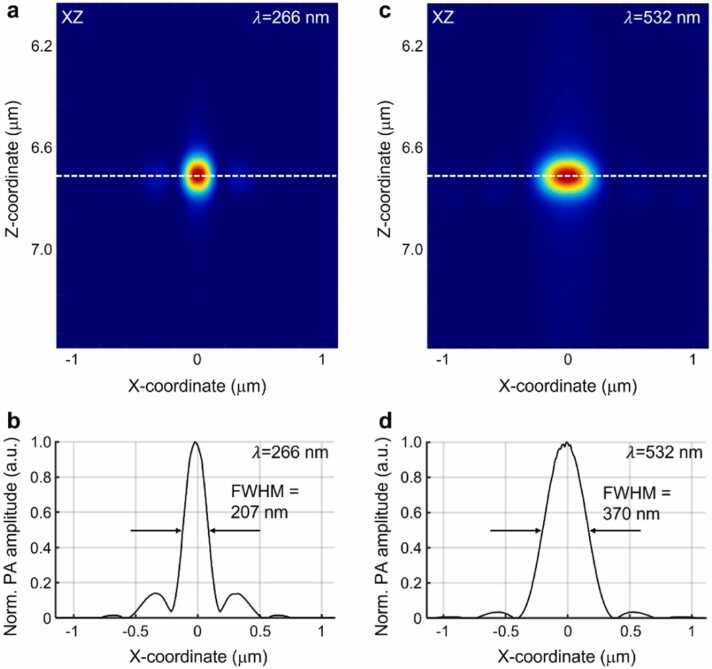
The 3D PA simulation results of a single cuboid target for the lateral resolution measurement. PA cross-section images of the single cuboid target excited by (a) UV and (c) visible light fields and (b, d) their normalized line profiles along the white dotted lines in (a) and (c), respectively.

A k-wave PA simulation was conducted to quantitatively assess the lateral resolution of the system. Using the simulation, a single cuboid target with a width of 45 nm was excited by both UV and visible light fields, resulting in the reconstruction of the PA B-scan images (Fig. 5a, c). In the case of the 266-nm UV light field, the PA signals exhibited weak side lobes surrounding the main lobe; this was attributed to the inherent properties of the light field (Fig. 5a). The FWHM value at 207 nm was calculated from the normalized line profile obtained along the white dashed line in Fig. 5b. Similarly, for the 532-nm visible light field, the PA B-scan image clearly depicted the cuboid target (Fig. 5c). Notably, the presence of considerably reduced side-lobe artifacts compared with the UV light field indicated the characteristic behavior of the numerical light field. The FWHM obtained from the normalized line profile along the white dashed line in Fig. 5d, was measured to be 370 nm. The results obtained from the k-wave PA simulation demonstrated that the lateral resolution of the system effectively captured the distinguishing features of the two excitations in the metalens-based configuration.

**Fig. 6:**
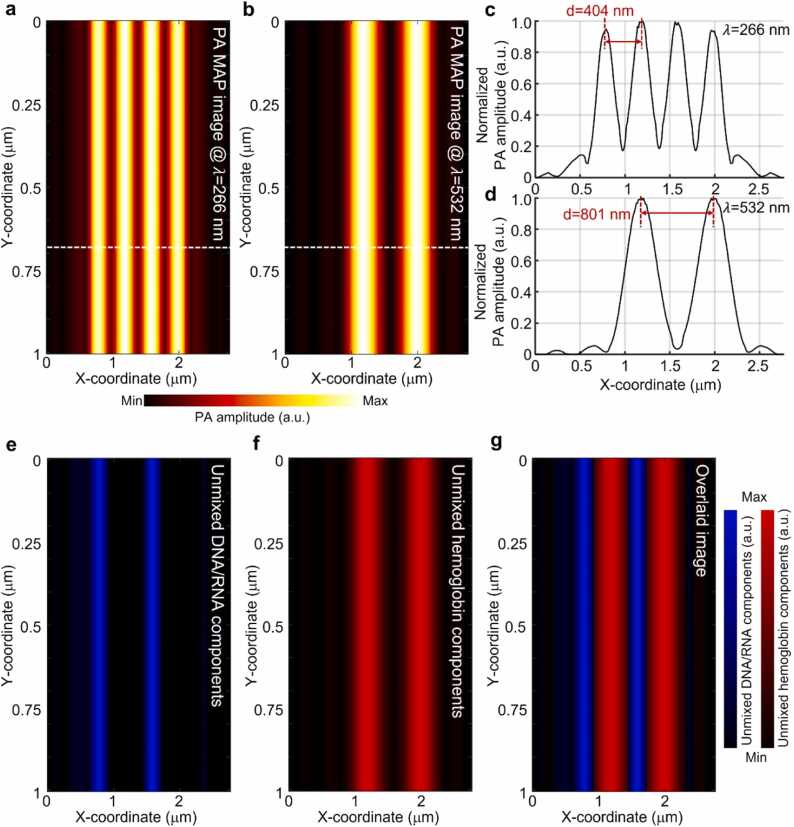
3D PA simulation results of DNA/RNA- and hemoglobin-mimicking phantoms. PA MAP images obtained by scanning DNA/RNA- and hemoglobin-mimicking phantoms (panel c in Fig. 3) with (a) UV and (b) visible light. (c, d) Normalized line profiles acquired along the white dashed lines in panels a and b, respectively. Spectrally unmixed (e) DNA/RNA and (f) hemoglobin from UV–visible PA MAP images, and (g) their overlaid component images. PA, photoacoustic; MAP, maximum amplitude projection; UV, ultraviolet.

The following simulation results were obtained to assess the feasibility of UV–visible PA imaging using a metalens. The phantom comprised four elongated cuboids that imitated the optical absorption properties of DNA/RNA and hemoglobin (Fig. 3c). Fig. 6a presents the obtained PA maximum amplitude projection (MAP) image using 266 nm excitation. As both DNA/RNA and hemoglobin exhibit strong light absorption at 266 nm, the four cuboids appeared undifferentiated in the image. Conversely, because of the considerably weaker absorption of DNA/RNA compared with that of hemoglobin at 532 nm, only hemoglobin-mimicking cuboids were identifiable in the PA MAP image acquired with 532 nm excitation (Fig. 6b). Although the four cuboids in the phantom were of equal size, the distinct beam sizes of the two excitations caused variations in the observed widths of the PA cuboids, as shown in Fig. 6a, b. Fig. 6c and d illustrate the normalized line profiles obtained along the white dashed lines in Fig. 6a and b, respectively. In the case of the 266 nm excitation, four spikes were observed, with a distance of d_1_ = 404 nm between adjacent peaks. This accurately reflected the intended spacing between the cuboids in the actual setup (Fig. 3c). For the 532 nm excitation, only the hemoglobin-mimicking cuboids were visible, and measured distance d_2_ was 801 nm; this was approximately twice the value of d_1_.

To distinguish between the DNA/RNA and hemoglobin components, we applied spectral unmixing techniques to the PA data obtained at two different wavelengths. Spectral unmixing is a powerful technique used in PA imaging to separate components based on their distinct spectral signatures. This allowed to identify and isolate specific components or targets within the acquired data by leveraging their unique spectral characteristics. In this case, the spectral unmixing process effectively separated the DNA/RNA and hemoglobin components, enabling us to visualize and analyze them individually. Fig. 6e and f show the results of extracting the unmixed DNA/RNA and hemoglobin components, respectively. We successfully extracted only the DNA/RNA component that was indistinguishable in the 266 nm PA image. Fig. 6g shows an overlay of unmixed DNA/RNA and hemoglobin components.

## 4 Discussion and conclusion

The ultimate direction of metalens-based UV–visible PAM lies in achieving high lateral resolution while miniaturizing the system. Conventional optical lenses often have limitations in downsizing the system due to their larger diameter and thickness. Additionally, commercially available UV–visible lenses typically have an NA that remains around 0.5. The metalens-based UV-Visible PAM we propose could serve as an alternative that fulfills the unmet needs of existing systems, offering the potential for both high lateral resolution and compact design. We proposed a numerical study of a dual-wavelength UV–visible ultra-compact metalens for PAM. The metalens with NA = 0.8, comprising two layers of HfO_2_ cylindrical nanopillar arrangements, exhibited diffraction-limited focal spots at wavelengths of 266 and 532 nm with a perfect focal length match.

For the PA simulation, we incorporated commercially available ultrasound transducer properties (40 MHz center frequency) into the k-Wave PA simulation. The PA simulations showed clear PA signal generation with lateral resolutions of 207 and 370 nm at wavelengths of 266 and 532 nm, respectively. DNA/RNA and hemoglobin phantoms with UV and visible light absorption bands were photoacoustically scanned in one focal plane, and the DNA/RNA and hemoglobin targets were clearly separated using a spectral unmixing technique. Through this, we demonstrated that metalens-based light fields can be practically employed for high-resolution PA imaging.

The advantages of the methodology comprised possibilities to integrate the metalens with portable multispectral PA devices owing to its extreme thinness of 1.4 µm and to perform PA imaging with different endogenous optical absorption contrasts with high spatial resolution. The NA of the metalens exceeded that of commercially available immersion-free objective lenses, making it an appealing cost-effective optical element for standard laboratory PA imaging of physiological slices. Furthermore, analogous design metalenses have the potential to be applied in UV lithography [44] or label-free single-protein detection based on UV autofluorescence [75], [76], [77], [78], [79], where a high NA and achromaticity in the UV region are vital. Finally, the presented metalens operation range can potentially be extended to longer wavelengths via multilayer stacking of metalens structure arrangements in the infrared range.

One important consideration is the integration of ultra-compact metalenses into the PAM system. There are several methods to install the lens in the PA imaging system: 1) By affixing the metalens to the end of an optical fiber, we enable PA excitation. Securing the optical fiber to a handheld or pen-type holder allows for the creation of a compact PAM imaging probe [59]. 2) In the recently developed near-field scanning PAM, a tapered fiber is brought close to the sample at tens of nanometers. To achieve this, the fiber is fixed to a tuning fork, and a shear-force mechanism is employed to maintain the precise distance. In our case, we can attach the small lens to the fiber, and then fix it to the tuning fork [5]. 3) The core diameter of an optical fiber used in intravascular PAM such as a catheter is hundreds of micrometers [80], [81]. In our case, a system can be constructed by attaching metalens to this optical fiber. Additionally, leveraging ultra-small metalenses opens exciting prospects for developing significantly smaller and thinner intravascular PAM devices [80], [81].

Reflection-mode PAM is considered more practical for in vivo imaging as compared to transmission-mode PAM. Moreover, it is worth noting that metalenses with their short focal lengths make the consideration of water immersion a reasonable choice for a reflection-mode PAM. The dielectric metalenses have been shown to operate in water environment for boosting NA of the optical system [82] or for underwater imaging [83]. Our metalens design could be applicable for this imaging condition as long as the top HfO_2_ pillar layer is protected by a SOG layer. Furthermore, reflection-mode PAM can be developed by combining miniaturized ultrasound transducers [84] or transparent ultrasound transducers [54], [59] with metalenses. In this case, the metalens focal length would have to increase beyond the thickness of a miniaturized ultrasound transducer, while increasing the size by the same factor would preserve high NA and spatial resolution. The simulations of light tailoring by larger metalenses could be performed via rigorous-coupled wave analysis which is a less computationally cumbersome method than FDTD. On the other hand, reflective metalenses would require operating in conjunction with bulky concave mirrors to guide the incident beam beyond the metalens. Moreover, the numerical aperture (NA) of reflective metalenses is typically below 0.6 [85]. Although reflective metalenses could provide larger phase modulation due to a longer propagation path inside resonators [86], they are still prone to chromatic aberrations.

Although we currently verified the potential of the metalens-based PAM through simulations, we anticipate experimental validation in the future with the practical development of metalenses. Furthermore, we believe that this advancement will significantly contribute to the downsizing of handheld, pen-type, and intravascular/endoscopic PAM systems [87].

## Declaration of Competing Interest

The authors declare that they have no known competing financial interests or personal relationships that could have appeared to influence the work reported in this paper.

## Data Availability

Data will be made available on request.
