# Supplementary material for: Dual-wavelength UV-visible metalens for multispectral photoacoustic microscopy: A simulation study
Source: Photoacoustics. 2023 Aug 16;32:100545. doi: 10.1016/j.pacs.2023.100545 (PMC10461252; doi:10.1016/j.pacs.2023.100545)
Supplement: Supplementary file 1 — Supplementary material [file mmc1.docx]

**Supplementary information for**

**Dual-Wavelength UV-Visible Metalens For Multispectral Photoacoustic Microscopy: A Simulation Study**

*Aleksandr Barulin,^1^ Hyemi Park,^2^ Byullee Park^1,^* and Inki Kim^1,2,^**

^1^ Department of Biophysics, Institute of Quantum Biophysics, Sungkyunkwan University, Suwon 16419, Republic of Korea

^2^ Department of Intelligent Precision Healthcare Convergence, Sungkyunkwan University, Suwon 16419, Republic of Korea

*Corresponding authors: byullee@skku.edu; [inki.kim@skku.edu](mailto:inki.kim@skku.edu)

Contents:

S1. Meta-atom height optimization

S2. Phase modulation of infrared light with 3-layer cascade

S3. Damage threshold discussion

**S1. Meta-atom height optimization**


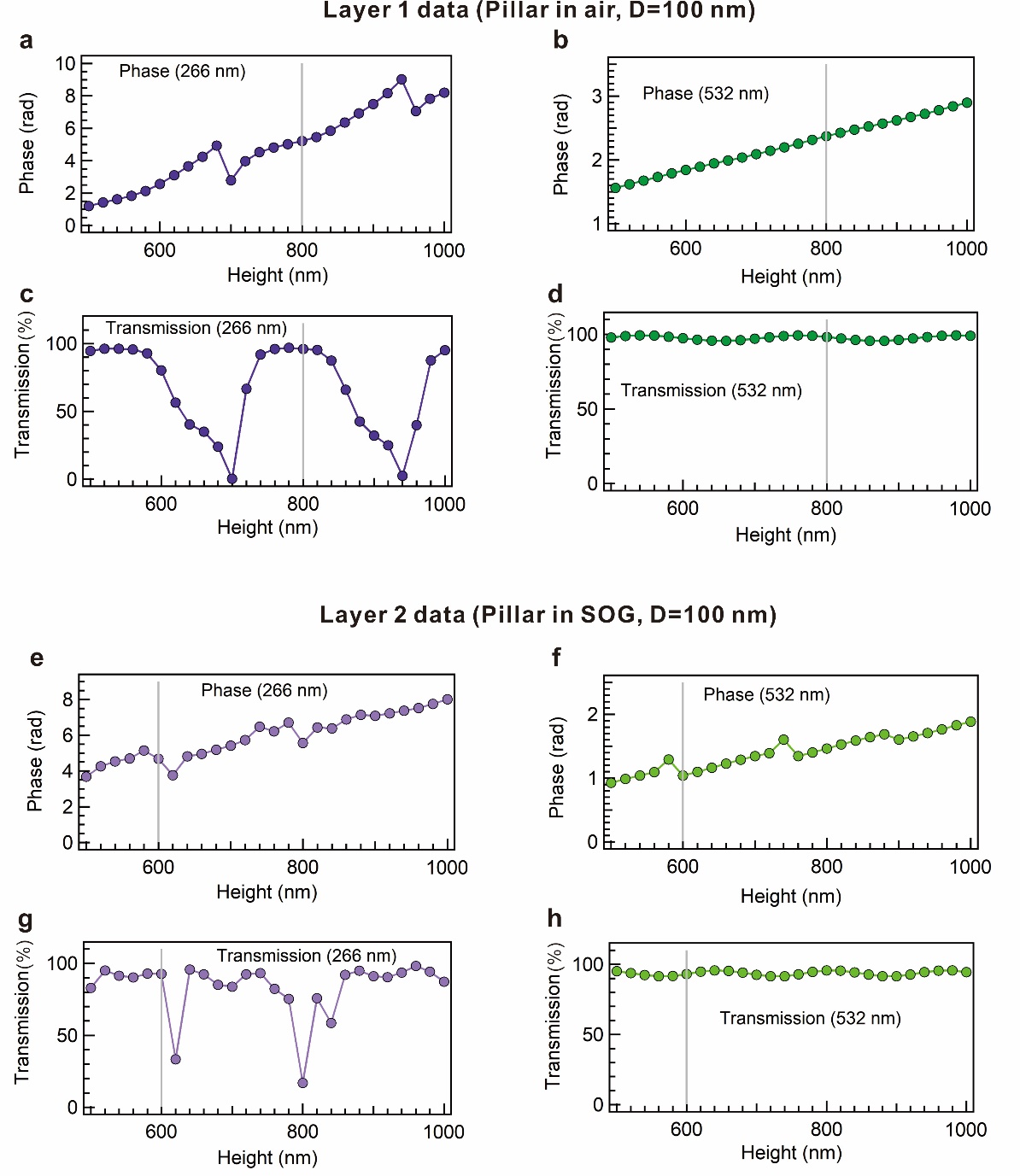


Figure S1. (a) and (b) Unwrapped phase data for various heights of a HfO_2_ pillar of 100 nm diameter in the air at 266 nm and 532 nm, respectively. (c) and (d) Transmission data for various heights of a HfO_2_ pillar of 100 nm diameter in the air at 266 nm and 532 nm, respectively. (e) and (f) Unwrapped phase data for various heights of a HfO_2_ pillar of 100 nm diameter in SOG at 266 nm and 532 nm, respectively. (g) and (h) Transmission for various heights of a HfO_2_ pillar of 100 nm diameter in SOG at 266 nm and 532 nm, respectively. The gray lines designate the selected heights for corresponding layers of the metalens.

**S2. Phase modulation of infrared light with 3-layer cascade**

**
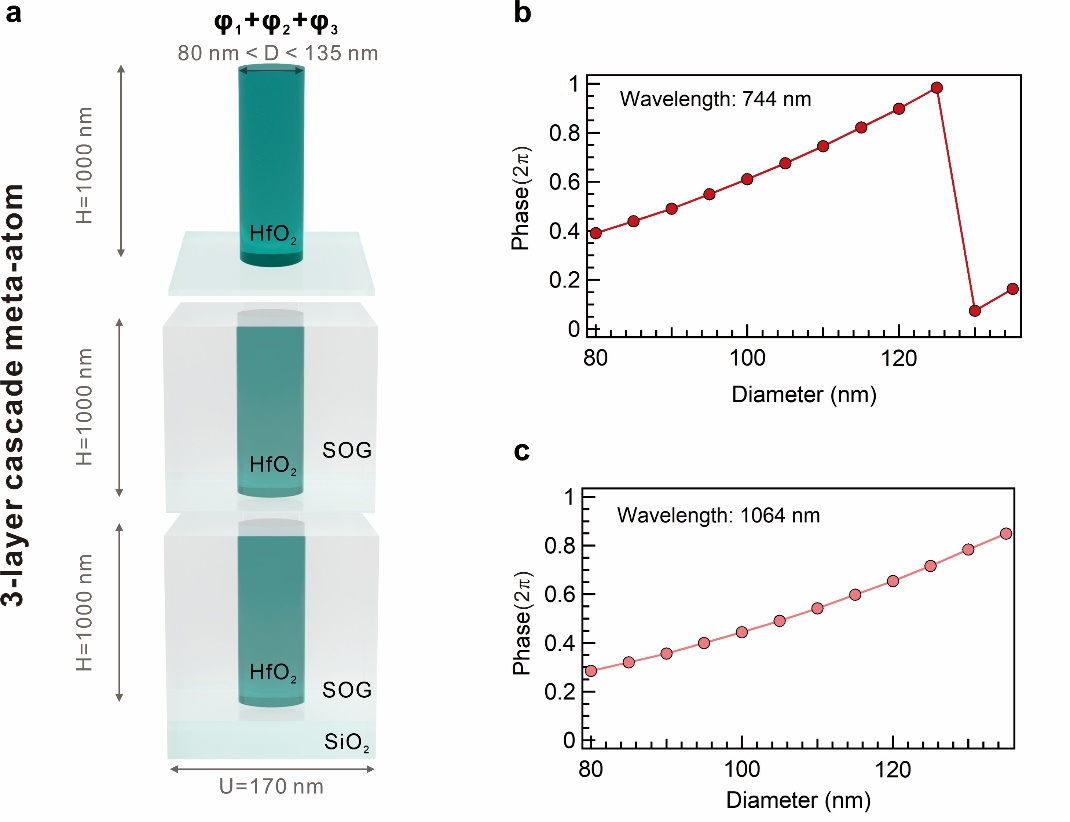
**

**Figure S2.** (a) Three-layer cascade meta-atom scheme for infrared phase modulation. For a demonstration of the maximal phase span that can be covered by the cascade, the height is fixed at 1 μm as the maximum feasible height for e-beam lithography in all three layers. (b) The wrapped phase dependence with the varying diameter of the cylinders at 744 nm. (c) The wrapped phase dependence with the varying diameter of the cylinders at 1064 nm. All cylinders are set to have identical diameters in the cascade.

**S3. Damage threshold discussion**

The theoretical estimation of the damage threshold is implemented following a recent theoretical report on the precise description of laser-induced thermal damage in thin films [S1]. The maximum temperature of the meta-atoms can be expressed as follows:

| $T_{max}(t)=T_{0}+\frac{\sqrt{2\pi}\cdot A_{f}(\lambda)\cdot E_{th}}{8\rho Ch\cdot\pi r_{0}^{2}}\left\{ erf\left[ \frac{\sqrt{2}(t-\delta t)}{\delta t} \right]+erf\left[ \sqrt{2} \right] \right\}$ | (S1) |
| --- | --- |

Here, T_max_ corresponds to the melting temperature of hafnium oxide (3031 K), *T_0_* is the temperature at the substrate interface which is fixed at 300 K, *A_f_*  is the absorption in the meta-atom cascade, *E_th_*  denotes the threshold energy, $\rho$ is the HfO_2_ density (9.68 g/cm^3^), *C* is the specific heat of HfO_2_ (120 J/(kg∙K)), *r_0_* is the radius of the metalens, *erf* is the error function ($erf \left( x \right)=\frac{2}{\sqrt{\pi}}\int_{0}^{x} e^{-t^{2}}dt$), $\delta t$ is the half width of a laser pulse duration. The absorption values at $\lambda$=266 nm and $\lambda$=532 nm have been retrieved from the FDTD simulations of a representative meta-atom cascade: D=115 nm for a pillar in SOG and D=110 nm for a pillar in air. We find the damage energy threshold at the time $t\gg\delta t$. The damage energy threshold amounts to 49 J/cm^2^ at 266 nm and 460 J/cm^2^ at 532 nm. By taking 10 ns as an example pulse width for the PAM studies, we retrieve the threshold peak power densities as 4.9 GW/cm^2^ and 46 GW/cm^2^ at corresponding wavelengths. These values lie in the proximity of a typical single-mode fiber damage threshold [S2]. As a side note, HfO_2_ is lossless and robust material. Its thin films in multilayered diffraction gratings were shown experimentally to heat up only by 3 K after a minute exposure to infrared laser of 1 kW [S3].

**Supplementary references**

[S1] G. Wang, J. Su, A new method to calculate the laser induced damage threshold of thin film, Optics Communications. 467 (2020) 125572.

[S2] Laser-Induced Damage in Silica Optical Fibers, (2023). https://www.thorlabs.com/newgrouppage9.cfm?objectgroup_id=1362.

[S3] I. Kim, S. So, J. Mun, K.H. Lee, J.H. Lee, T. Lee, J. Rho, Optical characterizations and thermal analyses of HfO2/SiO2 multilayered diffraction gratings for high-power continuous wave laser, Journal of Physics: Photonics. 2 (2020) 025004.
